# Investigating Normative Measurements of the Thumb and Index Finger to Aid in Reconstructive Surgery

**DOI:** 10.1016/j.jhsg.2025.100792

**Published:** 2025-07-23

**Authors:** Sydney Boike, Mikayla J. Baker, Lucas Ray, Ali Odenthal, Deb Bohn, Ann Van Heest

**Affiliations:** ∗ University of Minnesota Medical School, Minneapolis, MN; † Department of Orthopedic Surgery, University of Minnesota, Minneapolis, MN

**Keywords:** Anatomy, Congenital, Index, Reconstruction, Thumb

## Abstract

**Purpose:**

This study aims to investigate normative measurements of the thumb relative to the index finger; this may help guide hand surgery reconstruction and define a thumb as hyperplastic or hypertrophic.

**Methods:**

In total, 162 Minnesota State Fair participants were measured for thumb and index finger length, width, girth; joint range of motion (ROM) of the interphalangeal and metacarpophalangeal joints; and pinch and grip strength.

**Results:**

Participants’ age averaged 42.5 (range 14–88) years with 57% female, 86% White, and 86% right-handed. The right and left demonstrated similarity of thumb and index finger size for each participant. Men demonstrated larger length and girth of the thumb and index finger and stronger pinch and grip strengths but have minimal differences in ROM compared to women. The ratio of the index fingernail is 75% of the thumbnail. The length of the thumb is 73% of the index finger, and the thumb interphalangeal girth is 80% of the index finger proximal interphalangeal (PIP) joint. On average, the thumb tip sits 1.9 cm proximal to the index PIP joint.

**Conclusions:**

Anthropometric measurements of the thumb and index fingers demonstrate similarities of right and left hands for length, girth, ROM, and strength. Differences exist with size and strength greater for men compared with women, without differences in ROM. The thumb tip, on average, sits 19 mm proximal to the PIP joint, which is contrary to the conventional teaching for index pollicization to set the thumb tip length to the level of the adjacent finger PIP joint.

**Type of study/level of evidence:**

Therapeutic IV.

Approximately 1% to 2% of live births are born with congenital abnormalities, 10% of which are upper limb abnormalities.^1^^,^^2^ Abnormalities that affect the thumb make up 16% of these upper limb differences.^3^ Assessment of the thumb for hypoplasia or hyperplasia could assist the hand surgeon if normative data were available.

Previous studies by Sunil^4^ and Goldfarb et al^5^ were reviewed that investigate normative values of the thumb and index finger to aid in reconstruction. Both studies are limited in their sample size with 26 adults and 273 children in each study, respectively. This study was undertaken to apply their thumb and index finger measurement techniques to a larger adult population to provide more normative thumb and index finger size data, which can be used in reconstructive surgery.^6^

This study aims to better understand normative measurements of the thumb relative to the index finger. In doing so, we can better define parameters that would diagnose the degree to which a thumb as “hypoplastic” or “hypertrophic.” Furthermore, previous guidelines have indicated that for pollicization surgery, the thumb length is inset so that its tip is to the length of the index proximal interphalangeal (PIP) joint.^7^ Finally, we will use our results to expand upon, and contrast with, previous thumb size studies of Sunil^4^ and Goldfarb et al.^5^

## Materials and Methods

After institutional review board approval, participants were recruited and consented at a Minnesota State Fair research booth. The study received a “No greater than minimal risk” designation from the institutional review board, so informed oral consent was obtained. Normative thumb and index finger size data was gathered. Basic demographic information was collected, including age, male/female, race/ethnicity, and hand dominance.

Range of motion (ROM) of thumb metacarpophalangeal (MCP) and interphalangeal (IP) joints were obtained using a digit goniometer. Key and grip strengths were obtained using key pinch (Fabrication Enterprises, Vitality Medical) and grip dynamometers (Jamar, MDMaxx). Measurements and photos were recorded and stored securely in REDCap. The following measurements were obtained using rulers (Mr. Pen, Amazon): length of both thumbs, length of both index fingers, the width of the thumb fingernails, and width of index fingernails as described by Goldfarb et al.^5^ The girth of both thumbs at the joint level and the girth of both index fingers at the PIP joint level were measured using a finger circumference gauge (Lafayette Instrument). In order to minimize variation, three authors did all of the clinical measurements. Photographs of both hands were taken and used to measure the distance from the tip of the thumb to the index finger PIP joint; in order to minimize variation, two authors did all of these measurements.

Only skeletally mature thumbs and fingers were analyzed, i.e., girls starting at 14 years of age and boys starting at 16 years of age. Insufficient sample size was available to analyze separate race/ethnicity groups as Caucasians comprised 86% of the cohort. All statistical analyses were completed using Stata Statistical Software (18.0, Stata Corp LLC, College Station, TX). Descriptive statistics were calculated for each measurement category of interest described above. Differences among the various mean measurements of the right and left hand were compared using two-sample *t* tests. Similar methods were used to evaluate any if any differences existed between male and female study participants. For all data that is deemed statistically significant, the *P* value for all is <.05.

## Results

### Demographics

In total, 162 participants with 321 hands were included in the study. The average age was 42.5 years (range 14–88). Ninety-two participants were female, 70 were male. Eighty-six percent of the cohort (140) were White, 4% (7) were Asian, 3% (5) were multiracial, 2% (4) were Hispanic/Latinx, 2% (3) were Black, 1 was Egyptian, 1 was Native American, and 2 participants declined to answer. In total, 139 (86%) participants were right-handed, 18 (11%) were left-handed, and 5 (3%) were ambidextrous.

### Right compared with left

When comparing size measurements across all participants (Table 1), the right and left hands for each participant had no significant differences in thumb length, girth, or thumbnail width; the index finger had no significant differences in length, or fingernail width. There was a statistically significant difference (*P* < .05) between right and left index finger girth because of two outlier participants, with presumably little clinical difference as average size of right (5.0 cm) and left (5.2 cm) index finger girth was within 2 mm.

**Table 1:** Right Compared With Left Length Data

Measurement	Mean (cm)	SD	*P* Value
Thumb length			
Right (n = 161)	6.6	0.6	
Left (n = 160)	6.6	0.6	.624
Thumb girth			
Right	6.4	0.6	
Left	6.3	0.6	.146
Thumb fingernail width			
Right	1.6	0.2	
Left	1.6	0.2	.634
Index finger length			
Right	9.0	0.7	
Left	9.0	0.7	.585
Index finger girth			
Right	5.2	0.6	
Left	5.0	0.6	<.05∗
Index fingernail width			
Right	1.2	0.1	
Left	1.2	0.1	.939

∗ Indicates statistical significance.

Measurements of ROM of the right compared with left (Table 2) showed no differences between right and left for thumb MCP flexion, IP flexion, and IP extension. There was a statistically significant difference (*P* < .05) for thumb MCP extension with 5° of greater hyperextension on the left, which may be of questionable clinical significance.

**Table 2:** Right Compared With Left Range of Motion Data

Measurement	Mean (cm)	SD	*P* Value
MCP extension			
Right (n = 161)	−2.4	13.9	
Left (n = 161)	−7.4	14.7	<.05∗
MCP flexion			
Right	55.6	13.4	
Left	56.0	13.5	.762
IP extension			
Right	−22.5	13.1	
Left	−21.5	14.1	.360
IP flexion			
Right	70.4	15.1	
Left	73.2	14.5	.095

∗ Indicates statistical significance.

Measurements of strength of the right compared with the left (Table 3) showed no significant differences between right (average 17 lb) and left (average 17 lb) pinch strength. Grip strength was statistically significantly stronger on the right hand (average 81 lb) than the left hand (average 74 lb), presumably clinically significant because of the high incidence of right-hand dominance (*P* < .05).

**Table 3:** Right Compared With Left Strength Data

Measurement	Mean (cm)	SD	*P* Value
Key strength			
Right (n = 161)	17	5	
Left (n = 160)	17	5	.505
Grip strength			
Right	81	28	
Left	74	27	<.05∗

∗ Indicates statistical significance.

### Men compared with women

When analyzing size measurements based on men compared with women (Table 4), men had statistically significant larger measurements in all parameters: thumb length, girth, thumbnail width, as well as index finger length, girth and fingernail width (*P* < .05).

**Table 4:** Men Compared With Women Length Data

Measurement	Mean (cm)	SD	*P* Value
Thumb length			
Male (n = 67)	7.3	0.5	
Female (n = 92)	6.3	0.4	<.05∗
Thumb girth			
Male	6.8	0.4	
Female	5.9	0.4	<.05∗
Thumb fingernail width			
Male	1.8	0.1	
Female	1.50	0.1	<.05∗
Index finger length			
Male	9.5	0.5	
Female	8.7	0.5	<.05∗
Index finger girth			
Male	5.5	0.4	
Female	4.8	0.4	<.05∗
Index fingernail width			
Male	1.30	0.1	
Female	1.10	0.1	<.05∗

∗ Indicates statistical significance.

When analyzing ROM measurements for men compared to women (Table 5), women and men had no significant differences between right and left for thumb MCP flexion, IP flexion, and IP extension. There was a statistically significant difference (*P* < .05) for MCP extension with 6° of greater hyperextension in women.

**Table 5:** Men Compared With Women Range of Motion Data

Measurement	Mean (cm)	SD	*P* Value
MCP extension			
Male (n = 68)	−1.1	10.5	
Female (n = 92)	−7.90	11.8	<.05∗
MCP flexion			
Male	54.5	13.3	
Female	56.8	12.5	.271
IP extension			
Male	−20.3	12.6	
Female	−23.0	12.5	.181
IP flexion			
Male	72.7	14.3	
Female	71.3	13.3	.534

∗ Indicates statistical significance.

When comparing measurements of strength based on gender (Table 6), grip strength was statistically significantly stronger in men (average 20 lb) than in women (average 14 lb) (*P* < .05); pinch strength was statistically significantly stronger in men (average 101 lb) than in women (average 60 lb) (*P* < .05).

**Table 6:** Men Compared With Women Strength Data

Measurement	Mean (cm)	SD	*P* Value
Key strength			
Male (n = 67)	20	4	
Female (n = 92)	14	3	<.05∗
Grip strength			
Male (n = 68)	101	21	
Female (n = 91)	60	13	<.05∗

∗ Indicates statistical significance.

### Ratios

The ratio of the index fingernail width to thumbnail width was 0.75 (range 0.5–1.5). The ratio of thumb length to index finger length was 0.73 (range 0.52–0.93). The ratio of thumb girth at the IP joint to index finger girth at the PIP joint was 0.80 (range 0.6–1.09).

### Thumb tip distance to index PIP joint

As shown in the Figure, the distance from the tip of the thumb to the index PIP joint averaged 1.9 cm (range 0–3 cm), with no significant gender differences for women (average 1.8 cm) compared with men (average 2.0 cm).

**Figure:**
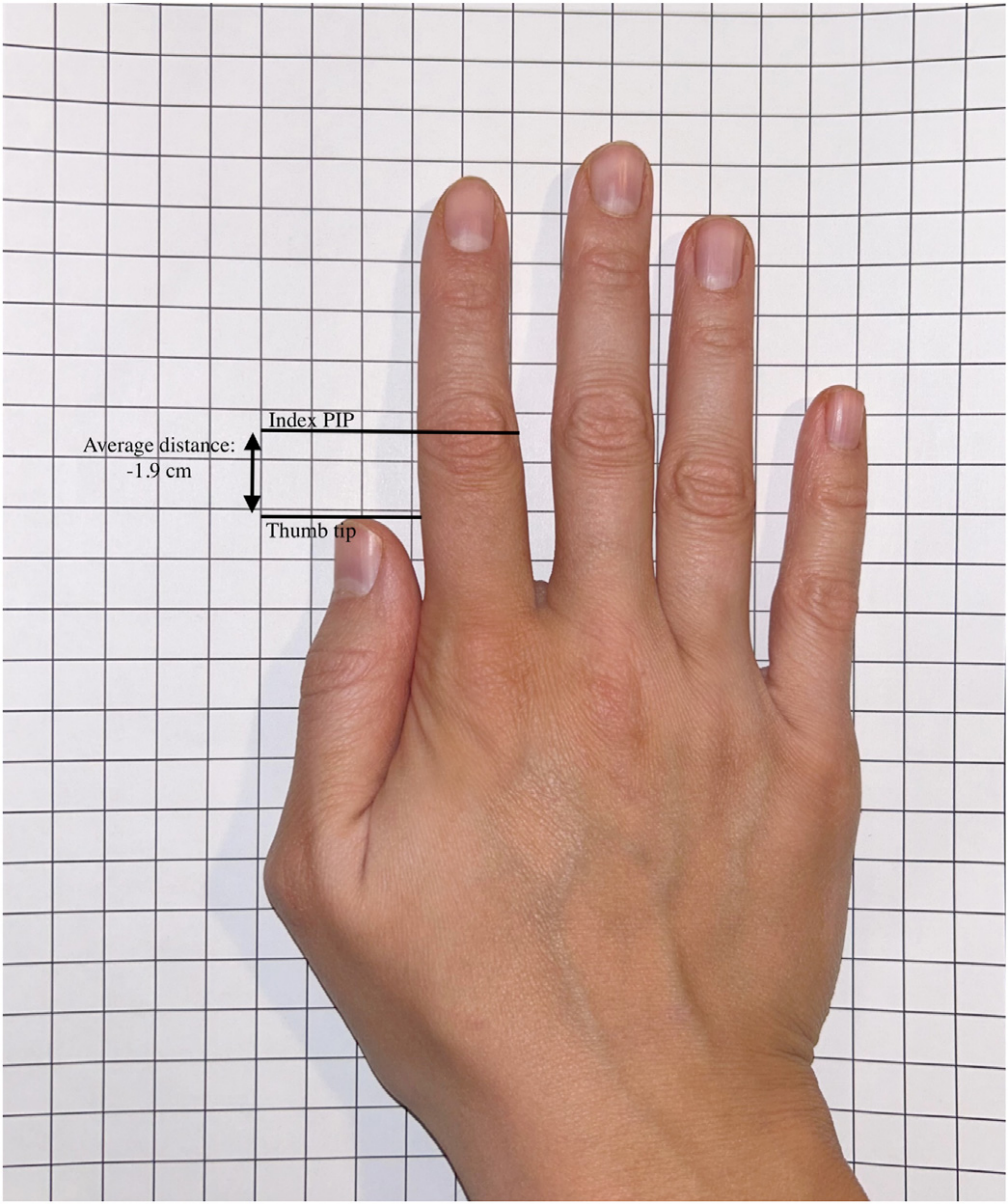
The distance from the tip of the thumb to the index finger PIP joint was measured for each subject as this is commonly used as a guide for setting thumb length during index pollicization surgery.

## Discussion

The study results demonstrate important anthropometric measurements regarding the thumb and index finger in 162 adult participants. First, the right and left hands demonstrate similarity of thumb and index finger size for each participant. Second, men have larger and stronger thumb and index finger, but have minimal differences in ROM compared to women. The ratio of the index fingernail is 75% of the thumbnail. The ratio of the length of the thumb is 73% of the index finger. The ratio of the thumb IP girth is 80% of the index finger PIP joint. We also reported that, on average, the thumb tip sits 1.9 cm proximal to the index PIP joint. This is contrary to the conventional teaching with the recommendation to set thumb length during index pollicization surgery so that the thumb tip is at the level of the adjacent finger PIP joint.

Other authors, including Goldfarb et al^5^ and Sunil,^4^ focused on understanding relationships between the thumb and index finger using multiple measurements. Measuring 273 pediatric patients, Goldfarb et al^5^ similarly found no differences in right compared with left thumb size and significant size differences based on gender. Because of the wide variations in hand size based on age, Goldfarb et al^5^ presented his results based on ratios. Although our study made direct measurements of thumb tip to index finger PIP joint averaging 1.9 cm, Goldfarb et al^5^ also reported the thumb length as shorter using ratios. Index fingernail width in the pediatric population was 67% of thumbnail, similar to our findings of 75%. Sunil^4^ measured ratios of thumb and index finger lengths in 26 adults and reported no significant differences in right compared with left. Sunil^4^ also reported no significant differences comparing thumb and index finger size ratios in men compared with women, although the sample size included only 10 women and 16 men. This is in contrast to our findings, which demonstrated that men have larger and stronger thumb and index finger, but have minimal differences in ROM, compared to women. The study by Sunil,^4^ however, reports on only 26 adult patients. Thus, our results of 162 adults expands the literature regarding thumb anthropometric measurements tremendously.

In reconstruction of the congenitally deficit thumb, an understanding of normal anthropomorphic thumb measurements provides a reconstructive goal. For example, in Nguyen et al,^8^ use of the toe to thumb transfer is inset to provide a “normal” length thumb; this study would assist in making that surgical plan. Additionally, use of index pollicization in congenital thumb hypoplasia could use this information for inset and length goals for the thumb reconstruction.

There are several areas that could represent limitations within this study. First, measurement variations may exist between examiners; inter-rater reliability was not measured. Second, some measurements were obtained, confirmed, or changed based on clinical photographs, which may add some variability. Third, this manuscript did not include skeletally immature participants in this data analysis, as Goldfarb et al^5^ had previously reported on 273 pediatric participants. Furthermore, this study reports primarily on Caucasian subjects and may not be applicable to other race/ethnicities. Recruitment occurred at a state fair venue, which may have introduced a bias for volunteer selection.

To conclude, the size and strength of the human thumb is critical for hand function. For hand surgeons, this article provides additional anthropometric measurements regarding normal adult thumb and index finger length and girth, nail width, and length comparisons.

## Statement of Informed Consent:

Informed consent was obtained from all individual participants included in the study.

## Statement of Human and Animal Rights:

All procedures followed were in accordance with the ethical standards of the responsible committee on human experimentation (institutional and national) and with the Helsinki Declaration of 1975, as revised in 2008.^4^ After institutional review board approval, participants were recruited and consented at a Minnesota State Fair research booth. The study received a “No greater than minimal risk” designation from the institutional review board, so informed oral consent was obtained from all study participants.

## Prior Presentations:

Podium presentation at Minnesota Orthopedic Society (MOS), Edina, MN, 5/03/24. Poster presentation at the International Federation of Societies for Surger of the Hand, Washington DC, USA 3/25.

## Conflicts of Interest

No benefits in any form have been received or will be received related directly to this article.
